# Decisions of the Dual-Channel Supply Chain under Double Policy Considering Remanufacturing

**DOI:** 10.3390/ijerph16030465

**Published:** 2019-02-05

**Authors:** Dingzhong Feng, Lei Ma, Yangke Ding, Guanghua Wu, Ye Zhang

**Affiliations:** College of Mechanical Engineering, Zhejiang University of Technology, Hangzhou 310023, China; fdz@zjut.edu.cn (D.F.); mlzjut@163.com (L.M.); dingyk@zjut.edu.cn (Y.D.); wgh20150326@zjut.edu.cn (G.W.)

**Keywords:** double policy, emission reduction, green consumers, remanufacturing, dual-channel supply chain

## Abstract

Considering the preference of green consumers for remanufactured products, a dual-sale-channel supply chain model with government non-intervention, government remanufacturing subsidy policy, and carbon tax policy is constructed, respectively. The difference of the optimal decision between the firm and the government under the two policies is discussed in this paper. Meanwhile, we analyze the influence of green consumers on the government’s optimal decision, based on social welfare maximization. It is found that without government intervention, social welfare is the lowest. The carbon tax policy is better when the proportion of green consumers and the environmental coefficient are extreme or moderate at the same time. Otherwise, the subsidy policy is better. The carbon tax policy is more effective than the subsidy policy in controlling carbon emissions. Profit-sharing contracts should be established by enterprises and governments to achieve win–win results.

## 1. Introduction

With economic growth racing ahead, environmental pollution and resource shortages have become increasingly prominent in recent years. Remanufacturing activities have attracted extensive attention and research for their great environmental and economic benefits [1]. Remanufacturing refers to a manufacturing process of the specialized repair or upgrading in order to transform remanufactured products to “like-new” [2]. Nowadays, cloud-based remanufacturing has been studied to achieve sustainable management of e-waste [3]. Different typologies of remanufacturing strategies are compared and evaluated from various perspectives [4,5,6]. As the ultimate form of recycling, remanufacturing is giving another option to the products [7].

To attain sustainable development of the environment, society, and economy, a range of environmental policies have been issued in many parts of the world to promote the remanufacturing industry. Carbon tax policy (CTP) and remanufacturing subsidy policy (RSP) are the two major regulations for accelerating the remanufacturing industry, and to control carbon emissions in the world. Subsidy policy holds a very important place in the development of remanufacturing, which is considered to be an impactful policy. Under this mechanism, the government motivates firms to produce remanufactured products through the provision of incentives. The Chinese government, for instance, has started to subsidize remanufacturers for each remanufactured truck engine sold [8]. Moreover, in order to curb carbon emissions, and to advance the harmonious development of the environmental economy, some punitive measures, for example, carbon tax policy, are also adopted by the government. That is, each unit of carbon emissions generated in the production process is taxed to the enterprise at a fixed tax rate. The European Union, Australia, and some countries in the Nordic region have adopted the carbon tax regulation [9,10]. The State Council has also promulgated the Regulations on the implementation of the Environmental Protection Tax Law of the People’s Republic of China, which came into effect on 1 January 2018 [11]. Hence, both RSP and CTP can greatly influence the enterprise’s operational behaviors and upgrade environmental standards.

Under RSP, manufacturers can gain a few more subsidies by expanding the sales of remanufactured products. Under CTP, the production of original products is an essential source of the manufacturer’s annual carbon expenditure. Hence, both RSP and CTP can promote manufacturers to make more environmentally friendly products by remanufacturing. Moreover, consumers are increasingly inspired and encouraged to purchase environmentally friendly products. Seventy five percent of Europeans were reported as being ready to purchase green products, even if they spent a little bit more [12]. A survey revealed that nearly 70% of Ningbo (Zhejiang province, China) residents prefer to pay for environmental improvement [13]. After implementing manufacturing, manufacturers should elaborate on an optimal operational strategy under double policy. Moreover, the government needs to determine which policies ought to be adopted, in order to maximize social welfare benefits.

There are few prior studies that examine the above challenges of the manufacturers and the government in remanufacturing, despite the significance of policy selection between RSP and CTP to the government, and the pricing and production decisions to the manufacturer under double policy. Our research aims to address this gap and to approach those challenges. Based on the consideration of environmentally friendly consumers, we establish a two-echelon dual-sale-channel supply chain for manufacturers to sell remanufactured products through electronic channels, and to sell ordinary products through independent retailers. We explore and examine the optimal operational strategies for manufacturers under the two policies. The government optimal strategies for unit carbon taxes and unit remanufacturing subsidies under RSP and CTP will be discussed as well. Furthermore, we compare and analyze the optimal social welfare outcomes under double policy, as well as presenting some managerial insights.

The reminder of this paper is organized as following. Section 2 reviews the relevant literature. In Section 3, we present the basic problem description and the model assumption. Section 4 investigates the optimal production and pricing problems under RSP and CTP. The double policies will be compared with regard to the maximum profit and carbon emissions, as well as social welfare benefits in Section 5. The numerical analysis presented in Section 6 illustrates several research results in this work. Finally, we summarize the main contents and propose some suggestions for future research directions in Section 7.

## 2. Literature Review

Considerable numbers of published papers that are related to remanufacturing subsidy policy and the carbon tax policy, in different respects, can be consulted in literature resources. The studies that are primarily relevant to this paper can be categorized into three streams: (1) an enterprise’s optimal production decisions under RSP; (2) an enterprise’s optimal production decisions under CTP; (3) comparison of an enterprise’s production decisions under the different government policies. 

### 2.1. An Enterprise’s Production Decisions under RSP

Mitra et al. [14] investigates the effects of subsidies upon promoting remanufacturing activity, and finds that subsidy sharing can motivate the manufacturers to design products that are more suitable for remanufacturing. Ma et al. [15] concentrates on the impacts of subsidies on the dual-channel supply chain, and the results show that both retailers and manufacturers can profit from the subsidy policy. Whether the e-retailer benefits or not is uncertain. To extend the study of [15] by considering the carbon emission qualities, Li et al. [16] analyzes three types of a supply chain, and finds that the subsidy strategy should be implemented only when the recycling price is within a certain range. Wang et al. [8] establishes system dynamics models to examines the effect of subsidy policy upon the development of the remanufacturing industry, and finds that the mixed-subsidy policy is superior to the single subsidy policy, but it involves much higher costs. Cohen et al. [17] studies subsidies for environmental protection technology adoption and the manufacturing industry’s response, and finds that subsidies provide a coordination mechanism. Li et al. [18] issues the role of subsidy policy in social welfare, and finds that the subsidy may be ineffective, and the reason for this phenomenon is analyzed as well. Shu et al. [19], considering the consumer willingness to pay and the competition to recycle, investigates the influence of government subsidies on recycling used products and engaging in remanufacturing for local manufacturers and nonlocal manufacturers. Fu et al. [20] compares the linear subsidy model and the fixed subsidy model, and explores the impacts of different subsiding policies on consumer surplus and social welfare. Zhao et al. [21] establishes a decision model, considering both the subsidy and the consumer’s acceptance for remanufactured products, and finds that the sharing ratio of the subsidy depends on the percentage of environmentally conscious consumers.

The above literature, except for [19,21], does not consider the effect of consumer preference, while it performs an important role in the enterprise’s production decisions. Furthermore, our research concentrates on the comparison of RSP and CTP, as well as taking account of social welfare, which is different from the earlier research. Given that it is essential for the government to select under the double policies, we adopt Stackelberg game theory to compare the social welfare results amongst the different policies, and propose several new managerial insights according to the main results of the study.

### 2.2. An Enterprise’s Production Decisions under CTP

Extensive attention is concentrated toward exploring the impact of carbon tax upon energy consumption or low-carbon technology, as exemplified by [22,23]. Here, we concentrate on studies of the production decisions under carbon tax policies. Chen et al. [24] investigates the sustainable production and pricing decisions of two competing enterprises under the carbon tax, and finds that the carbon tax imposed on low-efficiency enterprises should not be as much as high-efficiency enterprises. Xiao et al. [25] considers a two-echelon supply chain with price-sensitive demand under the carbon tax policy. It is shown that tax-sharing contracts can make the supplier and retailer benefit from the carbon footprint reduction. Yenipazarli et al. [26] characterizes the optimal carbon tax policy to achieve the inherent economic, social, and environmental profits of remanufacturing. Liu et al. [27] investigates the impacts of fairness concerns on production sustainability level under carbon tax policy. It is found that the carbon tax policy can motivate the manufacturer to improve the sustainable level of products. Wang et al. [28] considers the relationship between firm production decisions and government policies, as well as analyzing the influences of carbon tax on the decentralized supply chain and the centralized supply chain. Ma et al. [29] develops a dynamic programming model to study the influence of the carbon tax on calculating the optimal order quantity, and establishes the effective range of the carbon tax. Meng et al. [30] characterizes the optimal operational strategies, considering the effect of carbon tax, and finds that the ratio between environmental cost and the gross profit of the product can essentially affect the adjustment of the carbon tax rate. Wang et al. [31], considering a low-carbon emission technology investment, investigates the effects of a carbon emission tax on the optimal production decision of manufacturing and remanufacturing. Ding et al. [32] investigates service competition from the perspective of carbon tax policy, and concludes that imposing carbon tax alone cannot reduce carbon emissions. Saxena et al. [33] employs a modified cross-entropy solution method to resolve a supply chain planning model. They find that the model can assist governments in deciding on the carbon price.

As mentioned similarly in Section 2.1, few papers have considered the effects of consumer preference under CTP. In addition, social welfare functions, among the former studies, have not taken the adverse impact of carbon emissions on social welfare into consideration. Given that social welfare, to a great extent, is affected by carbon emission, it is thus important with regard to the social welfare function to take its negative consequences on society into account. In this research, we take account of the enterprise’s operational decisions, consumer’ preferences, and carbon emission reduction under double policies, as well as comparing the social welfare results under RSP and CTP, which complicates the issues further.

### 2.3. Comparison of an Enterprise’s Production Decisions under the Different Government Policies

An abundance of investigations compares different government policies, with full consideration of the influence of various factors, such as consumer surplus, carbon emissions, and so on. Our paper primarily concentrates on (i) the comparison of subsidy policies and carbon tax policies; and (ii) the comparison of cap-and-trade regulations, and carbon tax policies or subsidy policies.

By considering environmentally aware consumers, Bansal. [34] examines the welfare implications of subsidy policies and tax policies. It is found that optimal decisions rely on the magnitude of damage environmental parameters. Sheu et al. [35] analyzes the influence of subsidization and environmental taxation on green supply chain competition. The results show that chain-based profits and social welfare under equilibrium conditions increase by 306.6% and 27.8%, respectively. Shu et al. [36] investigates optimal production and pricing decisions under remanufacturing subsidies or carbon tax, and finds that both are beneficial to manufacturer. Yi et al. [37] studies the cooperation between upstream and downstream firms in emissions reduction and energy saving under subsidies and carbon tax policies. The results reveal that subsidy policies reduce the coordination efficiency, and the impact of carbon tax on the coordination efficiency depends on the initial carbon-emission level. In follow-up research, Yi et al. [38] examines the effects of carbon taxes and subsidies on enterprises’ operational decisions. It turns out that subsidies can reduce energy consumption and carbon emissions; yet this is not always the case for carbon taxes.

Some papers compare cap-and-trade regulations and subsidy policy or carbon tax policies in a macro-aspect, such as economic impact assessment [39], theory and experience analysis [40], and feasibility and desirability analysis [41]. Moreover, there are also many other studies that compare cap-and-trade policies and subsidy policies or carbon tax, based on micro-analysis. Cao et al. [42] studies the influences of cap-and-trade policies and subsidy policies upon the operations and related carbon emission reduction level. In that work, they discover that the optimal policy mostly rests with the environmental coefficient. Zakeri et al. [43] presents an analytical supply chain planning model to investigate the supply chain performance under cap-and-trade regulations and carbon tax policies, and finds that cap-and-trade can contribute to better supply chain performance. Xu et al. [44] studies the joint pricing and production issue with multiple products under carbon tax and cap-and-trade regulations. It is found that social welfare under cap-and-trade is not so as much as that under carbon tax policies. Drake et al. [45] studies the effect of cap-and-trade regulations and carbon tax upon an enterprise’s capacity decisions. They show that cap-and-trade contributes to a higher expected profit. Li et al. [46] examines production and transportation outsourcing issues under the cap-and-trade regulation, and the joint cap-and-trade and carbon tax regulation. The results indicate that the joint regulation is more effective for emissions reduction. Miao et al. [47] analyzes the optimal production and pricing strategies under the cap-and-trade program, and the carbon tax policy, and discovers that both can promote sales of remanufactured products, while reducing the demands of ordinary products.

The differences between our research and prior studies are described as the following. Our work introduces government environmental regulations and the consumer’s acceptance for remanufactured products in the two-echelon dual-channel supply chain, and investigates the impacts of RSP and CTP on production and pricing strategies. Furthermore, we examine the optimal strategies of the government under the double policies, and compare the social welfare results under RSP and CTP to propose the optimal policy. Combined with the theoretical analysis and the numerical simulation, we gain several meaningful managerial insights that are beneficial for the government to issue optimal policies, and meanwhile, for the firms to enhance competitiveness in environmental and economic performance.

## 3. Problem Description and Assumption

**Figure 1:**
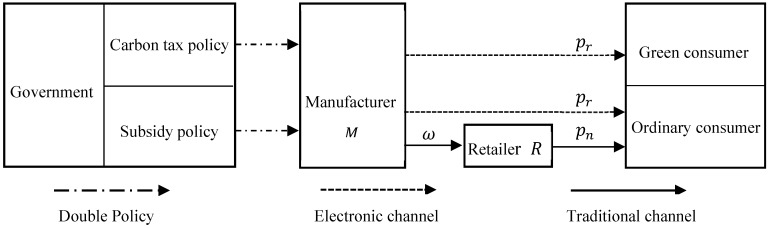
The dual-channel supply chain structure.

Combining with the observations and considerations from current practice, such as Dell [48] and Apple [49], they sell remanufactured products via their online store or electronic channels directly. At the same time, these manufacturers sell original products through independent retailers. Thus, this paper considers a dual-sale-channel supply chain consisting of a manufacturer who sells remanufactured products through electronic channels directly, and sells original products through independent retailers, as well as a retailer that only sells original products to consumers. This type of dual-channel has been widely studied [17,50,51]. Considering government regulations and green consumers, the decision-making framework on the dual-sale-channel supply chain is illustrated by Figure 1.

Motivated by the current practical conditions, the corresponding parameters and variables are denoted as the notations that are shown in Table 1. Meanwhile, we simplify the complicated conditions and propose some basic assumptions about the model.

### 3.1. The Demand Function

There are two types of consumers: ordinary consumers and green consumers. The proportion of green consumers is β. Following the example of earlier scholars [26,52], let coefficient δ∈U(0,1) refer to the consumer’s propensity to purchase the remanufactured products, while δ→0 denotes that the remanufactured products are hardly accepted, and δ→1 indicates that there are no consumers to choose the original product. Meanwhile, φ represents the consumer’s perceived value of original product, and φ is assumed to be uniformly distributed between 0 and 1. Considering that ordinary consumers have a demand for original products and remanufactured products, rather than just one product, the proportion of green consumers varies within a small range, and the value of the consumers’ acceptability is moderate.

Green consumers are of a higher education level and with low carbon awareness, considering that there is no quality difference between original products and remanufactured products, and they give priority to remanufactured products [53]. When purchasing remanufactured products through e-channels, the green consumer can obtain utility $U_{e}=\varphi -p_{r}$. An ordinary consumer can obtain utility $U_{cn}=\varphi -p_{n}$ and $U_{cr}=\delta \varphi -p_{r}$ via purchasing a new/remanufactured product through traditional/electronic channels, respectively. Based on the principle of utility maximization, the demand functions of the original products and remanufactured products are as follows:

(1) $$q_{n}=\left(1-\beta \right)\left[1-\left(p_{n}-p_{r}\right)/\left(1-\delta \right)\right]$$

(2) $$q_{r}=\beta \left(1-p_{r}\right)+\left(1-\beta \right)\left(\delta p_{n}-p_{r}\right)/\left[\delta \left(1-\delta \right)\right]$$

Note that we assume $p_{n}-1+\delta \leq p_{r}\leq \delta p_{n}$ to ensure the non-negativity of the demand functions.

### 3.2. The Cost Structure

The marginal costs of producing a remanufactured product and an original product are $c_{r}$ and c, respectively. Remanufactured products have a relative cost advantage, which has been widely-accepted in prior research [54,55]. Therefore, to ensure that building a remanufactured product is less costly than making an ordinary one, similar to Arya et al. and Xiong et al. [56,57], we can assume for the sake of argument that $c_{r}=0$. This is merely a convenient mathematical assumption.

Carbon emissions, without consideration for other processes, are mostly generated during the production process [58]. Let e be the carbon emissions for building one unit of original product. The total carbon emissions of these products are expressed as E; thus, $E=eq_{n}+\alpha eq_{r}$, where the coefficient α(0<α<1) is defined as the emission intensity of the remanufactured products. Similar to previous literature, such as in [26], we assume e=1. From the above, the total emissions can be expressed as $E=q_{n}+\alpha q_{r}$.

As described in [59], assuming that the environmental cost is rising in total carbon emissions, it can be represented by vE, where parameter v is the environmental coefficient that converts one unit of carbon emissions into one unit of currency. In addition, it is assumed that v≥0, which signifies that carbon emissions leave the social environment worse off.

### 3.3. The Game Order

We model the pricing and production decision-making as a Stackelberg game, in which the retailer and the manufacturer are the follower and the leader, respectively. Compared with the two enterprises (manufacturer and retailer), the government is the leader. That is, under a remanufacturing subsidy policy (carbon tax policy), the government first provides a prescribed subsidy amount (imposes a specified tax rate) for (on) the manufacturer. Secondly, the manufacturer determines its optimal production capacity and the price of remanufactured products and ordinary products, to maximize its anticipated profit. Finally, the retailer determines the retail price of ordinary products based on the best response of the manufacturer.

## 4. Model Formulation and Solution

In this section, we consider the double policy and government non-intervention policy, in which $\pi_{u}^{v}$ denotes the profit for player u under government policy v. Superscript v∈{Z,S,T} represents government non-intervention policy, RSP, and CTP, respectively, while subscript u∈{M,R} denotes the manufacturer and the retailer, respectively.

### 4.1. Model Z—Government Non-Intervention Policy

A benchmark for describing how government double policy affects the dual-sale-channel supply chain is given by Model Z.

Without government intervention, the retailer and manufacturer pursue their individual maximum profits in the process of the business decision. Backward induction is applied to this problem to achieve the equilibrium results of the Stackelberg game, in which the manufacturer is the leader and the retailer is the follower. The sequence of the game is as follows: The manufacturer first decides the wholesale price of the ordinary products, as well as the retail price of the remanufactured products, and then the retailer decides the retail price of ordinary products based on the manufacturer’s reaction function. Currently, the profit functions of the manufacturer and the retailer can be formulated as:

(3) $$\underset{p_{r},\omega_{n}}{\max\;}\Pi_{M}^{Z}=\left(1-\beta \right)\left(1-\frac{p_{n}-p_{r}}{1-\delta }\right)\left(\omega_{n}-c\right)+\left[\beta \left(1-p_{r}\right)+\frac{\left(1-\beta \right)\left(\delta p_{n}-p_{r}\right)}{\delta \left(1-\delta \right)}\right]\times p_{r}$$

(4) $$\underset{p_{n}}{\max\;}\Pi_{R}^{Z}=\left(1-\beta \right)\left(1-\frac{p_{n}-p_{r}}{1-\delta }\right)\left(p_{n}-\omega_{n}\right)$$

To improve readability, the optimal product price, production quantity, and profit are listed in Table A1 in Appendix A. Proofs are given in Appendix B.

### 4.2. Model S—Remanufacturing Subsidy Policy

In Model S, it is assumed that the government parameter μ is given. The manufacturer will receive a unit subsidy from the government for each unit of remanufactured product sold. The manufacturer wants to maximize profits by setting the optimal retail prices for remanufactured products and wholesale prices for ordinary products. The retailer sets optimal prices based on the manufacturer’s wholesale prices, and their profit is the same as Model Z. The manufacturer’s problem is:

(5) $$\underset{p_{r},\omega_{n}}{\max\;}\Pi_{M}^{S}=\left(1-\beta \right)\left(1-\frac{p_{n}-p_{r}}{1-\delta }\right)\left(\omega_{n}-c\right)+\left(p_{r}+\mu \right)\left[\beta \left(1-p_{r}\right)+\frac{\left(1-\beta \right)\left(\delta p_{n}-p_{r}\right)}{\delta \left(1-\delta \right)}\right]$$

The government decides on an optimal unit subsidy to maximize social welfare benefits according to the response function of the manufacturer. Similar to [20], the social welfare faced by government is denoted as the sum of the manufacturer’s profits, retailer’s profits, and the consumer surplus (*CS*), less the government expenditure and the environment cost. Therefore, the social welfare function can be presented as:

(6) $$\underset{\mu }{\max}\;SW^{S}=\Pi_{M}^{S}{}^{*}+\Pi_{R}^{S}{}^{*}+CS^{S}{}^{*}-\mu q_{r}^{S}{}^{*}-vE^{S}{}^{*}$$

where *CS* is composed of the ordinary product consumer surplus and the remanufactured product consumer surplus.

(7) $$CS=\left(1-\beta \right)\int_{p_{r}/\delta }^{\left(p_{n}-p_{r}\right)/\left(1-\delta \right)}\left(\delta \varphi -p_{r}\right)d\varphi +\beta \int_{p_{r}}^{1}\left(\varphi -p_{r}\right)d\varphi \;+\left(1-\beta \right)\int_{\left(p_{n}-p_{r}\right)/\left(1-\delta \right)}^{1}\left(\varphi -p_{n}\right)d\varphi$$

The optimal product price, production quantity, unit subsidy, and profit are also listed in Table A1 in Appendix A. Proofs are given in Appendix B.

**Proposition** **1.** *The impact of green consumers on the government’s optimal subsidy is as follows:*

When $0<v\leq v_{u}$, $\mu^{*}$ is increasing in β;

when $v_{u}<v$, $\mu^{*}$ is decreasing in β,

where $v_{u}=\frac{{\left(\delta -2\right)}^{2}-3\delta c}{2\delta \left(2-\alpha \right)}$.

The above relationships can be derived from the first-order partial conditions of algebraic computation. Proposition 1 shows that the environmental damage coefficient plays a significant part in the relationship between the optimal unit subsidies and the green consumers. When the environmental coefficient is not more than the critical value $v_{u}$, it indicates that the carbon emissions produced in the manufacturing process have a weak impact on the environment. The government should increase subsidies to stimulate market demand under the circumstance where green consumers gradually expand. On the contrary, the government should reduce unit remanufacturing subsidies as green consumers increase, to avoid the reduction of social welfare caused by the combined effect of soaring carbon emissions and high government fiscal expenditure.

### 4.3. Model T—Carbon Tax Policy

In this model, it is assumed that the government parameter t is given. The manufacturers are devoted to maximizing their own individual profits, while the government pursues the maximization of social welfare. The decision order of the supply chain is the same as that of Section 4.2. The retailer’s profit is the same as Model Z. The manufacturer’s problem is:

(8) $$\underset{p_{r},\omega_{n}}{\max\;}\Pi_{M}^{T}=\left(1-\beta \right)\left(1-\frac{p_{n}-p_{r}}{1-\delta }\right)\left(\omega_{n}-c-t\right)+\left(p_{r}-\alpha t\right)\left[\beta \left(1-p_{r}\right)+\frac{\left(1-\beta \right)\left(\delta p_{n}-p_{r}\right)}{\delta \left(1-\delta \right)}\right]$$

The consumer surplus is the same as that of Model S. The government decision-making goal remains to maximize social welfare. However, the government decision, currently, is presented as:

(9) $$\underset{t}{\max}SW^{T}=\Pi_{M}^{T}{}^{*}+\Pi_{R}^{T}{}^{*}+CS^{T}{}^{*}+\left(t-v\right)E^{T}{}^{*}$$

The optimal product price, production quantity, unit carbon tax, and profit are still listed in Table A1 in Appendix A. Proofs are given in Appendix B.

**Proposition** **2.** *Under CTP, the optimal carbon tax is decreasing in green consumers.*

The above relationships can be derived from the first-order partial conditions of the algebraic computation. Proposition 2 shows that, compared with the subsidy policy, the relationship between the carbon tax policy and the green consumer will not be affected by the environmental coefficient. The increase in green consumers enlarges the market demand. Although lower carbon taxes have reduced the government’s fiscal revenues, increased supply chain profits and rising consumer surpluses make up for the decline in social welfare, due to reduced taxes. A high proportion of green consumers and carbon tax policy that directly controls carbon emissions will both promote environmental quality. When there are more green consumers, the government will impose a lower carbon tax; instead, carbon tax should be increased to ensure the maximization of social welfare. This indicates that green consumers can enable the government to improve environmental quality, and to increase social welfare on the premise of reducing carbon tax. 

## 5. The Comparison of the Two Policies

We compare the optimal operational decisions, total carbon emissions, and enterprise’s profit, as well as the social welfare benefits, under the remanufacturing subsidy policy and the carbon tax policy.

**Proposition** **3.** *The relationship between the two total carbon emissions is as follows:*

(a) *When*$\frac{\delta }{2-\delta }<\alpha <1$, *then*$E^{S}{}^{*}>E^{Z}{}^{*}>E^{T}{}^{*}$;
(b) *When*$\;0<\alpha <\frac{\delta }{2-\delta }$, $\frac{t}{\mu }>\frac{\delta -\left(2-\delta \right)\alpha }{\left(2-\delta \right)\alpha^{2}-2\alpha \delta +\delta }$,
  *If*$\beta_{a}<\beta <1$, *then*$E^{S}{}^{*}>E^{Z}{}^{*}>E^{T}{}^{*}$,
  *If*$0<\beta \leq \beta_{a}$, *then*$\;E^{T}{}^{*}\leq E^{S}{}^{*}\leq E^{Z}{}^{*}$,
(c) *When*$\;0<\alpha <\frac{\delta }{2-\delta }$, $\frac{t}{\mu }\leq \frac{\delta -\left(2-\delta \right)\alpha }{\left(2-\delta \right)\alpha^{2}-2\alpha \delta +\delta }$,
  *If*$\beta_{b}<\beta <1$, *then*$E^{S}{}^{*}>E^{Z}{}^{*}>E^{T}{}^{*}$,
  *If*$0<\beta \leq \beta_{b}$, *then*$\;E^{S}{}^{*}\leq E^{T}{}^{*}\leq E^{Z}{}^{*}$,
  Where $\beta_{a}=\frac{\delta -2\alpha +\alpha \delta }{\delta -2\alpha +3\alpha \delta -2\alpha \delta^{2}}$, $\beta_{b}=\frac{\left(\delta -2\alpha +\alpha \delta \right)\left(\mu +\alpha t\right)+\delta \left(\alpha -1\right)t}{\left(\delta -2\alpha +3\alpha \delta -2\alpha \delta^{2}\right)\left(\mu +\alpha t\right)+\delta \left(\alpha -1\right)t}$.

Proposition 3(a) says that when the emission intensity is higher than a threshold; that is, when the remanufactured product fails to meet certain environmental standards, the carbon emissions under RSP are the greatest in the three models, while that under CTP is the least. The difference in carbon emissions in the three models is mainly affected by the remanufactured products in the case of high carbon emission intensity. 

Proposition 3 shows that a higher carbon tax, relative to unit subsidy, may make the carbon emissions lower than that under RSP, while the impact of a higher subsidy on carbon emissions control, relative to the unit carbon tax, also depends on the green consumer and emissions intensity. Carbon tax policy that directly controls carbon emissions is not affected by emissions intensity. Both green consumers and carbon tax policy can directly affect carbon emissions. Even if the carbon tax is relatively low, more carbon emissions can be reduced, with more green consumers and low emission intensities. Only if the emission intensity is lower than a threshold, that is, when the remanufactured product reaches a certain level of environmental protection, will the subsidy policy for the indirect control of carbon emissions produce relatively less carbon emissions in the case of less green consumers.

Proposition 3 indicates CTP under high pressures is always superior to RSP, regardless of the environmental protection level of the remanufactured products. Only when the market implements high environmental protection grade remanufactured products and when the government carries out high subsidy policies can RSP be better than CTP.

**Proposition** **4.** *The optimal original product price and the optimal remanufactured product price satisfy the following relationship*: $p_{n}^{S^{*}}<p_{n}^{Z^{*}}<p_{n}^{T^{*}}$, $p_{r}^{S^{*}}<p_{r}^{Z^{*}}<p_{r}^{T^{*}}$.

The above relationships can be derived directly from the algebraic comparison. Proposition 4 shows that the price of original products and remanufactured products in Model T is the highest, and that of two products in Model S is the lowest, which indicates that different policies of the government will cause different trends in the prices of the original products and the manufactured products. When the government implements the RSP, the manufacturer will transfer half of the unit subsidy to consumers by cutting the price of the remanufactured products. This decision promotes the sales of remanufactured products while restraining the demand for ordinary products. To gain more competitive advantages, retailers have to choose to lower the price of the ordinary product. In order to reduce the loss of profits caused by the government carbon tax policy, the manufacturers will transfer part of the unit carbon tax to the retailer and consumers by increasing the wholesale price of ordinary products and the retail price of remanufactured products, respectively, so that the price of remanufactured products under this policy is the highest. Meanwhile, retailers have to put up the prices of original products accordingly, to ease the rise in wholesale price by the manufacturer. Therefore, the price of ordinary products under this policy is also the highest.

**Proposition** **5.** *The optimal original product quantity and the optimal profit of the retailer satisfies the following relationship:*

(a) *When*μ>(1−α)t, *then*$q_{n}^{S^{*}}<q_{n}^{T^{*}}<q_{n}^{Z^{*}}$, $\Pi_{R}^{S}{}^{*}<\Pi_{R}^{T}{}^{*}<\Pi_{R}^{Z}{}^{*}$;
(b) *When*μ≤(1−α)t, *then*$q_{n}^{T^{*}}\leq q_{n}^{S^{*}}<q_{n}^{Z^{*}}$, $\Pi_{R}^{T}{}^{*}\leq \Pi_{R}^{S}{}^{*}<\Pi_{R}^{Z}{}^{*}$.

The above relationships can be derived simply from the algebraic comparison. Proposition 5 shows that a higher remanufacturing subsidy, relative to the unit carbon tax, will make the demand for original products and retailer’s profit the lowest, under this policy. However, the relatively lower unit subsidy will result in a higher demand for original products and the retailer’s profit, than that under CTP. No matter which policy is implemented, the demand for ordinary products and the retailer’s profit are the greatest without government intervention. According to the analysis of Proposition 4, the subsidy policy reduces the demand for ordinary products, while the levy of the carbon tax causes the retailer to raise the price of ordinary products, and inevitably reduces their demand. Therefore, the demand for ordinary products is the largest when the government does not intervene. The higher the unit subsidy, the less the demand will be for ordinary products under this policy. On the contrary, the demand for ordinary products is higher under RSP. The retailer’s unit profit also satisfies the above rule, that is, under a higher subsidy, $p_{n}^{S^{*}}-\omega_{n}^{S^{*}}<p_{n}^{T^{*}}-\omega_{n}^{T^{*}}<p_{n}^{Z^{*}}-\omega_{n}^{Z^{*}}$; under a higher carbon tax, $p_{n}^{T^{*}}-\omega_{n}^{T^{*}}<p_{n}^{S^{*}}-\omega_{n}^{S^{*}}<p_{n}^{Z^{*}}-\omega_{n}^{Z^{*}}$. So, the retailer’s profit presents the above relationship.

Proposition 5 indicates that government intervention will reduce the retailer’s margin, and that the extent of the decline depends on the degree of government intervention. Currently, the retailer’s enthusiasm to sell ordinary products will be dampened. The profit of the manufacturer is also affected by the green consumers, and the size relationship will be given in the example.

**Proposition** **6.** *The size relationship between social welfare could be explored as seen below:*

(a) *When*$v_{1}<v<v_{2}$,
  *If*$\beta_{1}<\beta <\beta_{2}$, *then*$SW^{Z}{}^{*}<SW^{S}{}^{*}<SW^{T}{}^{*}$;
  *If*$\beta <\beta_{1}$, or $\beta >\beta_{2}$, *then*$SW^{S}{}^{*}>SW^{T}{}^{*}>SW^{Z}{}^{*}$.
(b) *When*$v<v_{1}$, or $v>v_{2}$,
  *If*$\beta_{1}<\beta <\beta_{2}$, *then*$SW^{S}{}^{*}>SW^{T}{}^{*}>SW^{Z}{}^{*}$;
  *If*$\beta <\beta_{1}$, or $\beta >\beta_{2}$, *then*$SW^{Z}{}^{*}<SW^{S}{}^{*}<SW^{T}{}^{*}$.

Proposition 6 shows that the size relationship between social welfare in three supply chain models depends on the green consumers and the environmental coefficient. Compared with the situation without government intervention, the social welfare under RSP or CTP is larger, which is intuitive. The social welfare under double policy presents the following relationship. When the environmental coefficient is between the critical value, the social welfare under RSP, relative to CTP, decreases first and then increases as the number of green consumers increases. When the environmental coefficient is greater than $v_{2}$ or less than $v_{1}$, the social welfare under RSP, relative to CTP, with the increase of green consumers, is reduced after the first increase.

Proposition 6 indicates that when society imposes stricter emissions requirements on corporations, to avoid the high economic losses caused by carbon emissions, the government will give priority to CTP. With the further expansion of green consumers, the carbon tax policy is gradually replaced by RSP. Currently, green consumers effectively control carbon emissions, which requires a subsidy policy to stimulate consumer demand to improve social welfare. When green consumers reach a certain scale (i.e., $\beta =\beta_{2}$), the CTP gradually surpasses the subsidy policy. In this case, the government’s carbon tax revenues bring about the largest improvement in social welfare. When emission requirements for enterprises are within a certain range (i.e., $v_{1}<v<v_{2}$), the government should first choose the subsidy policy, and preferentially expand the market demand in the case where the environmental coefficient is not high. When green consumers reach the threshold ($\beta_{1}$), the carbon tax policy can bring more social welfare. While green consumers break through the threshold ($\beta_{2}$), which means that there are more environmentalists, an incentive subsidy policy is more likely to promote social welfare maximization than CTP. Therefore, the government should adopt appropriate policies based on the social requirements for enterprise carbon emissions and green consumers.

## 6. Numerical Analysis

In this section, we conduct the following numerical analysis of decision-making obtained in the different policies. Considering that it is difficult to gain reliable and complete data from the manufacturing companies, relevant parameters are designed to better illustrate the above theoretical results, and to explain some managerial insights. The parameters of the example are set as the following: market demand scale Q=1000, and the unit production cost c=100. For the convenience of comparative analysis, it is assumed that the proportion of green consumers, emission intensity, environmental damage coefficient, and the consumer’s propensity to buy the remanufactured products are 0.6, 0.5, 0.8, and 0.5, respectively. The optimal decision-making, profit and corresponding social welfare values in the three dual-sale-channel supply chain models are shown in Table 2 (where t=151.3514, μ=155.0000).

According to the results in Table 2, the carbon emissions under CTP are not so much as those under RSP. After calculation, the emission intensity threshold α=1/3<1/2; that is, the remanufactured products cannot reach a certain level of environmental protection. Therefore, the punitive carbon tax policy plays a more effective role than the subsidy policy in controlling carbon emissions, which is in agreement with Proposition 3 in Section 5.

The price of remanufactured products and original products under CTP is the highest, while the price of them is the lowest under RSP. As the dominant leader of the game, the manufacturer shares part of its subsidy with the consumer by selling remanufactured products through electronic channels under RSP, while the carbon tax caused by CTP will be transferred to the retailer and the consumers through the wholesale price and the retail price of remanufactured products, respectively. It implies that different policies will result in different changes in the prices of original products and remanufactured products, which coincides with Proposition 4 in Section 5.

Under the double policy, the demand for original products is reduced, and meanwhile, the profits of the retailer are impaired. The retailer’s profit and the demand for original products under RSP are the lowest. This indicates that government intervention increases the market share of remanufactured products. After calculation, μ>t/2, and the subsidy policy currently produces a more effective and efficient outcome. By comparing the data, it is not difficult to notice that the growth of the manufacturer’s profit under RSP is much more than the decline of the retailer’s profit. Due to environmental effects, the high carbon tax paid by the manufacturer under CTP leads to a simultaneous reduction in profits for both the retailer and the manufacturer. The loss can be compensated by forming a reasonable profit-sharing contract between the government and the members of the supply chain. This is in accordance with Proposition 5 in Section 5.

Green consumer consciousness is a critical market force, which can constrain the environmental performance of enterprises [60]. When the government implements different policies on the supply chain, the green consumers have a major impact upon the government decision-making, and they will indirectly influence the decisions of the supply chain and social welfare. Conventional studies less related to the contents of this idea. This paper, taking the RSP and CTP implemented by the government for the manufacturer as an example, analyzes the impact of green consumers on government decision-making parameters, the ecological environment, and social welfare.

Table 3 indicates that as the number of green consumers expands, the optimal unit subsidy rises, while the optimal carbon tax decreases. Currently, the threshold of the environmental coefficient $v_{u}=1.3125>0.8$, which indicates that the government stimulates demand through subsidies. Although carbon emissions under CTP continue to rise, they are always lower than those under RSP and government non-intervention policies. This is consistent with Propositions 1 and 2 in Section 5.

With the increasing number of green consumers, social welfare under government non-intervention and double policy are improved to varying degrees. It shows that society should encourage consumers to enhance their environmental awareness. Social welfare under government non-intervention is always lower than that under RSP or CTP, which indicates that the government’s intervention has further promoted social welfare, and that the implementation of double policy is necessary. When the proportion of green consumers is less than 0.45, the social welfare under RSP is not so much as that under CTP; otherwise, the social welfare under RSP is higher than that under CTP. Currently, the threshold of the environmental coefficient and the proportion of green consumers are $v_{1}=0.0875$, $v_{2}=0.5247$, $\beta_{1}=0.4940$, $\beta_{2}=1.7495$. This is consistent with Proposition 6 in Section 5. Therefore, it is particularly important to formulate different strategies according to the green consumers.

Considering that the manufacturer’s profit is affected by many factors, the comparison process under different policies is more complex and difficult to prove by an analytical method. For discussion purposes, we only vary the proportion of green consumers. Through a trend curve, we attempt to explore the relationship between the manufacturer’s profits under RSP and CTP, as well as government non-intervention policy.

**Figure 2:**
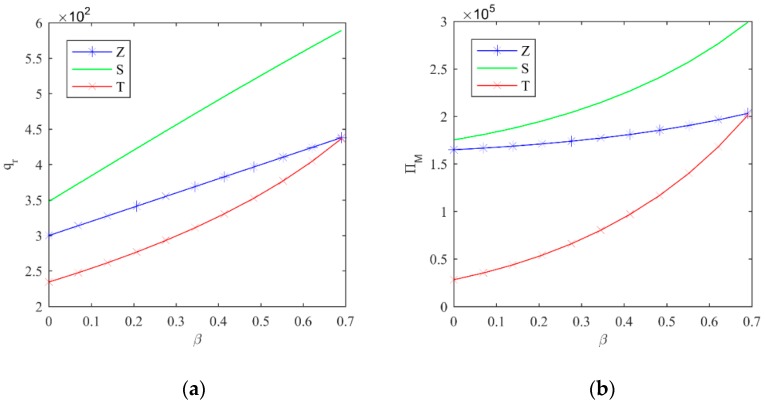
(**a**) Trend of $q_{r}$ with β; and (**b**) trend of $\Pi_{M}$ with β.

Figure 2a demonstrates that, with the increase of green consumers, the demand for remanufactured products in the three models will expand. The number of remanufactured products under CTP is the least, while that under RSP is the largest. It indicates that green consumers can support the market share of remanufactured products, and consequently, the government should enhance the consumer’s awareness of environmental protection and knowledge of remanufactured products through national education. Obviously, Figure 2b shows that in the three models, manufacturers’ profits will increase as the number of green consumers increases. The profits of manufacturers under government non-intervention are lower than those under RSP, but higher than those under CTP. This indicates that a profit-sharing mechanism should be formed between the government and enterprises, to compensate for the loss of the manufacturer’s profit. According to the previous analysis, under RSP, manufacturer should also establish profit-sharing contracts with the retailer, so as to avoid channel conflicts that are caused by the retailer’s depressed enthusiasm to sell ordinary products. 

## 7. Conclusions

Considering the preferences of green consumers for remanufactured products, we analyzed the decisions of production and pricing in a dual-sale-channel supply chain under RSP and CTP. The optimal decisions and profits were derived, based on Stackelberg game theory, in the three models. In the end, we compared carbon emissions, the optimal decisions, and enterprise’s profits in the context of double policies, as well as exploring the influence of the relevant coefficient upon dual-sale-channel supply chain decisions, carbon emissions, profits, and social welfare. Several significant managerial insights were obtained as follows:

(1) Both the remanufacturing subsidy policy and the carbon tax policy can improve social welfare. The government should employ CTP when the proportion of green consumers and the environmental coefficient are within a certain range at the same time. Otherwise, the government should issue RSP.
(2) Compared with government non-intervention, under RSP, the price and demand of the original products are lower. The price of remanufactured products is lower, while the demand is higher. The profits of the retailer are impaired. The profits of the remanufacturers are increased, and the increase of the manufacturer’s profits is always more than the decrease of the retailer’s profits. Under CTP, the demand for original products decreases, and the price increases. The price of remanufactured products increases, and the demand decreases. The profits of the retailer and manufacturer are impaired at the same time, and the government revenue increases. Therefore, the three parties can set reasonable profit-sharing contracts to ensure the stability of the supply chain, and to achieve a win-win situation for both the government and members of the supply chain.
(3) The government can implement either one of a double policy to reduce carbon emissions, and a carbon tax policy that directly control emissions is more effective than RSP.
(4) When the environmental coefficient is less than (greater than) a threshold, the government’s optimal unit subsidy should rise (decrease) as the number of green consumers increases. The government should reduce the carbon tax with the increase of green consumers.

A few limitations and shortages exist in the course of this research. However, some of them may point out the fields and potential for further research. Firstly, this paper focuses on the operational strategies of a single remanufacturer, and it is worthwhile to examine the situation in which multiple competing manufacturers exist under double policy. Similarly, this research could be extended from manufacturers to other members of the supply chain. Second, the government subsidizes the manufacturers per unit of remanufactured product sold. In practice, consumers of remanufactured products may also receive government subsidies. Thus, how the government allocates subsidies needs further study. Third, we do not analyze the profit-sharing contract between the members of the supply chain and the government in detail; however, it may be a fruitful avenue for follow-up research work.

## Figures and Tables

**Table 1:** Parameters and variables.

Notation	Descriptions
$q_{r}$, $q_{n}$	Production quantity or customer demand of the remanufactured products and the original products respectively
$p_{r}$, $p_{n}$	Unit retail price of the remanufactured products and original products, respectively
ω	Unit wholesale price of the remanufactured products
c	Cost of manufacturing an original product
Q	The primary market size
δ	Consumer’s propensity to buy the remanufactured products
φ	Consumer’s perceived value of the original product
e	Unit of carbon emissions
α	Emission intensity of the remanufactured products
v	Environmental damage coefficient
E	Total emissions
β	Ratio of the green consumer
μ	Unit remanufacturing subsidy
t	Unit carbon tax

**Table 2:** The optimal decisions and profits in the different policies.

	Model Z	Model S	Model T
$\omega_{n}$	657.1429	657.1429	732.8185
$p_{n}$	757.1429	718.3929	810.1158
$p_{r}$	357.1429	279.6429	387.4131
$q_{n}$	80.0000	49.0000	61.8378
$q_{r}$	420.0000	559.5000	395.7838
E	248.0000	272.8000	220.1514
$\Pi_{R}$	8000	3001	4779.9
$\Pi_{M}$	194,570	270,480	159,140
CS	136,140	176,600	120,040
SW	140,310	145,120	141,160

**Table 3:** The influence of green consumers when the government implements the double policies.

β	$\mu^{*}$	$t{\;}^{*}$	$CS^{Z\;}{}^{*}$	$CS^{S\;}{}^{*}$	$CS^{T\;}{}^{*}$	$E^{Z\;}{}^{*}$	$E^{S\;}{}^{*}$	$E^{T}{\;}^{*}$	$SW^{Z\;}{}^{*}$	$SW^{S\;}{}^{*}$	$SW^{T\;}{}^{*}$
0.05	38.7629	630.2277	79,690	88,000	22,690	314.0000	318.0701	107.9155	13,700	14,150	37,680
0.10	45.9574	602.5751	86,630	96,670	31,250	308.0000	313.0553	118.7914	25,810	26,430	46,960
0.15	53.6264	572.8285	93,300	105,240	39,820	302.0000	308.1670	129.5786	37,830	38,650	56,250
0.20	61.8182	540.7407	99,670	113,700	48,400	296.0000	303.4182	140.2667	49,760	50,810	65,550
0.25	70.5882	506.0241	105,710	122,040	57,010	290.0000	298.8235	150.8434	61,570	62,890	74,850
0.30	80.0000	468.3417	111,410	130,250	65,650	284.0000	294.4000	161.2945	73,270	74,910	84,180
0.35	90.1266	427.2966	116,730	138,340	74,360	278.0000	290.1671	171.6031	84,840	86,850	93,540
0.40	101.0526	382.4176	121,630	146,280	83,150	272.0000	286.1474	181.7495	96,270	98,700	102,930
0.45	112.8767	333.1412	126,060	154,090	92,070	266.0000	282.3671	191.7095	107,550	110,460	112,370
0.50	125.7143	278.7879	130,000	161,740	101,150	260.0000	278.8571	201.4545	118,670	122,120	121,870
0.55	139.7015	218.5304	133,380	169,250	110,440	254.0000	275.6537	210.9495	129,590	133,680	131,460
0.60	155.0000	151.3514	136,140	176,600	120,040	248.0000	272.8000	220.1514	140,310	145,120	141,160
0.65	171.8033	75.9857	138,220	183,790	130,030	242.0000	270.3475	229.0065	150,810	156,430	151,010

* denotes the optimal operational decisions and results under the optimal decisions.

## Appendix A

**Table A1:** Comparison of the optimal decisions under different policies.

	Model Z	Model S	Model T
$\omega_{n}$	$\frac{c+1}{2}+Y$	$\frac{c+1}{2}+Y$	$\frac{c+t+1}{2}+Y$
$p_{r}$	$\frac{\delta }{2}+Y$	$\frac{\delta -\mu }{2}+Y$	$\frac{\delta +\alpha t}{2}+Y$
$p_{n}$	$\frac{3-\delta +c}{4}+Y$	$\frac{3-\delta +c-\mu }{4}+Y$	$\frac{3-\delta +c+\left(1+\alpha \right)t}{4}+Y$
$q_{n}$	$\frac{\left(1-\beta \right)\left(1-\delta -c\right)}{4\left(1-\delta \right)}$	$\frac{\left(1-\beta \right)\left(1-\delta -c-\mu \right)}{4\left(1-\delta \right)}$	$\frac{\left(1-\beta \right)\left[1-\delta -c-\left(1-\alpha \right)t\right]}{4\left(1-\delta \right)}$
$q_{r}$	$\frac{\left(1-\beta \right)\left(1-\delta +c\right)}{4\left(1-\delta \right)}+\frac{\beta }{2}$	$\frac{\left(1-\beta \right)\left[\left(1-\delta +c\right)\delta +\left(2-\delta \right)\mu \right]}{4\delta \left(1-\delta \right)}+\frac{\beta \left(1+\mu \right)}{2}$	$\frac{\left(1-\beta \right)\left[\delta \left(1-\delta +c\right)+t\left(\delta -2\alpha +\alpha \delta \right)\right]}{4\delta \left(1-\delta \right)}+\frac{\beta \left(1-\alpha t\right)}{2}$
$\Pi_{R}$	$\frac{\left(1-\beta \right){\left(1-\delta -c\right)}^{2}}{16\left(1-\delta \right)}$	$\frac{\left(1-\beta \right){\left(1-\delta -c-\mu \right)}^{2}}{16\left(1-\delta \right)}$	$\frac{\left(1-\beta \right){\left[1-\delta -c-\left(1-\alpha \right)t\right]}^{2}}{16\left(1-\delta \right)}$
$\Pi_{M}$	$\left(1-\beta \right)\left[\begin{array}{c} \frac{\left(1-c\right)\left(1-\delta -c\right)}{8\left(1-\delta \right)}+\\ \frac{\delta \left(1-\delta +c\right)}{8\left(1-\delta \right)}+\frac{Y}{2} \end{array}\right]+$ $\frac{\beta }{2}\times \left(Y+\frac{\delta }{2}\right)$	$\left(1-\beta \right)\left[\begin{array}{c} \frac{\left(1-c\right)\left(1-\delta -c-\mu \right)}{8\left(1-\delta \right)}-\frac{Y^{2}}{\delta }+\\ \frac{\left(\delta +\mu \right)\left[\mu \left(2-\delta \right)+\delta \left(1-\delta +c\right)\right]}{8\delta \left(1-\delta \right)} \end{array}\right]+$ $\beta \left(Y+\frac{\delta +\mu }{2}\right)\left(1-Y-\frac{\delta -\mu }{2}\right)$	$\left(1-\beta \right)\left[\begin{array}{c} \frac{\left(1-c-t\right)\left[1-\delta -c-\left(1-\alpha \right)t\right]}{8\left(1-\delta \right)}-\frac{Y^{2}}{\delta }+\\ \frac{\left(\delta -\alpha t\right)\left[\delta \left(1-\delta +c\right)+t\left(\delta +\alpha \delta -2\alpha \right)\right]}{8\delta \left(1-\delta \right)} \end{array}\right]+$ $\beta \left(Y+\frac{\delta -\alpha t}{2}\right)\left(1-Y-\frac{\delta +\alpha t}{2}\right)$
μ/t	/	$\frac{D_{1}}{\left(1-\beta \right)\left(4-3\delta \right)+4\beta \delta \left(1-\delta \right)}$	$\frac{-\left(D_{2}\beta +D_{3}\right)}{\left[\left(4\delta^{2}-7\delta +4\right)\beta +3\delta -4\right]\alpha^{2}+\delta \left(\beta -1\right)\left(1-2\alpha \right)}$
	$D_{1}=\left(\beta -1\right)\left(3\delta c+4\delta v-8\alpha v+4\alpha \delta v\right)+\delta \left(\delta -1\right)\left(3\beta -8\alpha \beta v+1\right)$		
	$D_{2}=3\delta \left(\alpha -1\right)\left(\delta -1+c\right)+4v\left[\left(2\delta^{2}-3\delta +2\right)\alpha^{2}-2\alpha \delta +\delta \right]$		
	$D_{3}=4v\left[\left(2-\delta \right)\alpha^{2}-2\alpha \delta +\delta \right]-3\delta \left(1-c-\delta +\alpha c\right)+\alpha \delta \left(\delta -1\right)$;Y=δβ(1−δ)/[2(1−β+δβ)]		

## Appendix B

Equilibrium solutions of the supply chain under different policies are calculated and presented in the following context below.

### Appendix B.1. Solutions under Model Z

For the given wholesale price $\omega_{n}$ and the retail price of remanufactured products $p_{r}$, we take the first-order partial derivative of $\Pi_{R}^{Z}$ with respect to the retail price of the original products $p_{n}$, then we can obtain $\partial \Pi_{R}^{Z}/\partial p_{n}=\left(1-\beta \right)\left(1-\delta +\omega_{n}+p_{r}-2p_{n}\right)/\left(1-\delta \right)$.

Since the second-order derivatives $\partial^{2}\Pi_{R\;}^{Z}/\partial p_{n}{}^{2}=-2\left(1-\beta \right)/\left(1-\delta \right)<0$, the profit of the retailer is a strictly concave function with respect to the retail price $p_{n}$, and then we have the best retailer’s response $p_{n}^{Z}=\left(\omega_{n}^{Z}+1-\delta +p_{r}^{Z}\right)/2$ by equating the first-order condition to zero. Substituting the value of $p_{n}^{Z}$ into the manufacturer’s profit function, we get:

$\frac{\partial \Pi_{M}^{Z}}{\partial p_{r}}=\frac{1}{2\delta \left(1-\delta \right)}\left[2p_{r}\left[\left(1-\beta \right)\left(\delta -2\right)-2\beta \delta \left(1-\delta \right)\right]+\delta \left(1-\delta \right)\left(1+\beta \right)+\delta \left(1-\beta \right)\left(2\omega_{n}-c\right)\right]$,

$\frac{\partial \Pi_{M}^{Z}}{\partial \omega_{n}}=\frac{1}{2\left(1-\delta \right)}\left(1-\beta \right)\left(c-\delta +1-2\omega_{n}+2p_{r}\right)$.

Then, we take the second-order partial derivatives of $\Pi_{M}^{Z}$ with respect to $p_{r}$ and $\omega_{n}$, and we can obtain the Hessian matrix.

$\left[\begin{array}{cc} \frac{\partial^{2}\Pi_{M}^{Z}}{\partial p_{r}{}^{2}} & \frac{\partial^{2}\Pi_{M}^{Z}}{\partial p_{r}\partial \omega_{n}}\\ \frac{\partial^{2}\Pi_{M}^{Z}}{\partial \omega_{n}\partial p_{r}} & \frac{\partial^{2}\Pi_{M}^{Z}}{\partial \omega_{n}{}^{2}} \end{array}\right]=\left[\begin{array}{cc} \frac{\left(1-\beta \right)\left(\delta -2\right)}{\delta \left(1-\delta \right)}-2\beta & \frac{1-\beta }{1-\delta }\\ \frac{1-\beta }{1-\delta } & \frac{\beta -1}{1-\delta } \end{array}\right]$

We assume $A_{1}=\left|\frac{\partial^{2}\Pi_{M}^{Z}}{\partial p_{r}{}^{2}}\right|$, $A_{2}=\left|\begin{array}{cc} \frac{\partial^{2}\Pi_{M}^{Z}}{\partial p_{r}{}^{2}} & \frac{\partial^{2}\Pi_{M}^{Z}}{\partial p_{r}\partial \omega_{n}}\\ \frac{\partial^{2}\Pi_{M}^{Z}}{\partial \omega_{n}\partial p_{r}} & \frac{\partial^{2}\Pi_{M}^{Z}}{\partial \omega_{n}{}^{2}} \end{array}\right|$, where $A_{1}=\frac{\left(1-\beta \right)\left(\delta -2\right)}{\delta \left(1-\delta \right)}-2\beta <0$, $A_{2}=\frac{2\left(1-\beta \right)\left(1-\beta +\beta \delta \right)}{\delta \left(1-\delta \right)}>0$. In other words, the Hessian matrix is negative definite.

Therefore, $\Pi_{M}^{Z}\left(p_{r},\omega_{n}\right)$ is a concave function of $p_{r}$ and $\omega_{n}$. Let $\partial \Pi_{M}^{Z}/\partial p_{r}=0$ and $\partial \Pi_{M}^{Z}/\partial \omega_{n}=0$, and then we can obtain the optimal decisions $\omega_{n}^{Z}{}^{*}=\left(c+1\right)/2+Y$ and $p_{r}^{Z}{}^{*}=\delta /2+Y$. Substituting the above values into the retailer’s best response, we get $p_{n}^{Z}{}^{*}=(3-\delta +c)/4+Y$. This completes the proof.

### Appendix B.2. Solutions under Model S

The retailer’s profit is the same as Model Z, and we have the best retailer’s response $p_{n}^{S}=\left(\omega_{n}^{S}+1-\delta +p_{r}^{S}\right)/2$. Substituting the value of $p_{n}^{S}$ into the manufacturer’s profit function, we get:

$\begin{array}{c} \frac{\partial \Pi_{M}^{S}}{\partial p_{r}}=\frac{1}{2\delta \left(1-\delta \right)}[2p_{r}\left[\left(1-\beta \right)\left(\delta -2\right)-2\beta \delta \left(1-\delta \right)\right]+\delta \left(1-\delta \right)\left(1+\beta \right)+\delta \left(1-\beta \right)\left(2\omega_{n}-c\right)+\\ \mu \left[\left(1-\beta \right)\left(\delta -2\right)-2\beta \delta \left(1-\delta \right)\right]], \end{array}$

$\frac{\partial \Pi_{M}^{Z}}{\partial \omega_{n}}=\frac{1}{2\left(1-\delta \right)}\left(1-\beta \right)\left(c-\delta +1-2\omega_{n}+2p_{r}+\mu \right)$.

Then, we take the second-order partial derivatives of $\Pi_{M}^{S\;}$ with respect to $p_{r}$ and $\omega_{n}$, and we can obtain the Hessian matrix.

$\left[\begin{array}{cc} \frac{\partial^{2}\Pi_{M}^{S\;}}{\partial p_{r}{}^{2}} & \frac{\partial^{2}\Pi_{M}^{S}}{\partial p_{r}\partial \omega_{n}}\\ \frac{\partial^{2}\Pi_{M}^{S}}{\partial \omega_{n}\partial p_{r}} & \frac{\partial^{2}\Pi_{M}^{S\;}}{\partial \omega_{n}{}^{2}} \end{array}\right]=\left[\begin{array}{cc} \frac{\left(1-\beta \right)\left(\delta -2\right)}{\delta \left(1-\delta \right)}-2\beta & \frac{1-\beta }{1-\delta }\\ \frac{1-\beta }{1-\delta } & \frac{\beta -1}{1-\delta } \end{array}\right]$.

We assume $B_{1}=\left|\frac{\partial^{2}\Pi_{M}^{S\;}}{\partial p_{r}{}^{2}}\right|$, $B_{2}=\left|\begin{array}{cc} \frac{\partial^{2}\Pi_{M}^{S\;}}{\partial p_{r}{}^{2}} & \frac{\partial^{2}\Pi_{M}^{S}}{\partial p_{r}\partial \omega_{n}}\\ \frac{\partial^{2}\Pi_{M}^{S}}{\partial \omega_{n}\partial p_{r}} & \frac{\partial^{2}\Pi_{M}^{S\;}}{\partial \omega_{n}{}^{2}} \end{array}\right|$, where $B_{1}=\frac{\left(1-\beta \right)\left(\delta -2\right)}{\delta \left(1-\delta \right)}-2\beta <0$, $B_{2}=\frac{2\left(1-\beta \right)\left(1-\beta +\beta \delta \right)}{\delta \left(1-\delta \right)}>0$. In other words, the Hessian matrix is negative definite.

Therefore, $\Pi_{M}^{S}\left(p_{r},\omega_{n}\right)$ is a concave function of $p_{r}$ and $\omega_{n}$. Let $\partial \Pi_{M}^{S}/\partial p_{r}=0$ and $\partial \Pi_{M}^{S}/\partial \omega_{n}=0$, and we can obtain the optimal decisions $\omega_{n}^{S}{}^{*}=\left(c+1\right)/2+Y$ and $p_{r}^{S}{}^{*}=\left(\delta -\mu \right)/2+Y$. Substituting the above values into the retailer’s best response, we get $p_{n}^{S}{}^{*}=(3-\delta +c-\mu )/4+Y$. From (6), we get

$\frac{\partial SW^{S}\left(\mu \right)}{\partial \mu }=\frac{\delta \left(1-\delta \right)\left(3\beta +1\right)+3\delta \left(1-\beta \right)c+4v\left[\delta \left(1-\beta \right)+\left[\left(\delta -2\right)\left(1-\beta \right)-2\beta \delta \left(1-\delta \right)\right]\alpha \right]+\left(4\beta +3\delta -7\beta \delta +4\beta \delta^{2}-4\right)\mu }{16\delta \left(1-\delta \right)}$.

$\frac{\partial^{2}SW^{S}\left(\mu \right)}{\partial \mu^{2}}=\left(1-\beta \right)\frac{3\delta -4}{16\delta \left(1-\delta \right)}-\frac{\beta }{4}$.

Since the second-order derivatives $\partial^{2}SW^{S}\left(\mu \right)/\partial \mu^{2}<0$, $SW^{S}\left(\mu \right)$ is a concave function of μ. Let $\frac{\partial SW^{S}\left(\mu \right)}{\partial \mu }=0$, and we can obtain the optimal unit subsidy

$\mu^{*}=\frac{\left(\beta -1\right)\left(3\delta c+4\delta v-8\alpha v+4\alpha \delta v\right)+\delta \left(\delta -1\right)\left(3\beta -8\alpha \beta v+1\right)}{\left(1-\beta \right)\left(4-3\delta \right)+4\beta \delta \left(1-\delta \right)}$.

### Appendix B.3. Solutions under Model T

The retailer’s profit is the same as Model Z, and we have the best retailer’s response $p_{n}^{T}=\left(\omega_{n}^{T}+1-\delta +p_{r}^{T}\right)/2$. Substituting the value of $p_{n}^{T}$ into the manufacturer’s profit function, we get

$\begin{array}{c} \frac{\partial \Pi_{M}^{T}}{\partial p_{r}}=\frac{1}{2\delta \left(1-\delta \right)}[2p_{r}\left[\left(1-\beta \right)\left(\delta -2\right)-2\beta \delta \left(1-\delta \right)\right]+\delta \left(1-\delta \right)\left(1+\beta \right)+\delta \left(1-\beta \right)\left(2\omega_{n}-c-t\right)-\\ \alpha t\left[\left(1-\beta \right)\left(\delta -2\right)-2\beta \delta \left(1-\delta \right)\right]], \end{array}$

$\frac{\partial \Pi_{M}^{T}}{\partial \omega_{n}}=\frac{1}{2\left(1-\delta \right)}\left(1-\beta \right)\left[c-\delta +1-2\omega_{n}+2p_{r}+t\left(1-\alpha \right)\right]$.

Then we take the second-order partial derivatives of $\Pi_{M}^{T\;}$ with respect to $p_{r}$ and $\omega_{n}$, and we can obtain the Hessian matrix.

$\left[\begin{array}{cc} \frac{\partial^{2}\Pi_{M}^{T\;}}{\partial p_{r}{}^{2}} & \frac{\partial^{2}\Pi_{M}^{T}}{\partial p_{r}\partial \omega_{n}}\\ \frac{\partial^{2}\Pi_{M}^{T}}{\partial \omega_{n}\partial p_{r}} & \frac{\partial^{2}\Pi_{M}^{T\;}}{\partial \omega_{n}{}^{2}} \end{array}\right]=\left[\begin{array}{cc} \frac{\left(1-\beta \right)\left(\delta -2\right)}{\delta \left(1-\delta \right)}-2\beta & \frac{1-\beta }{1-\delta }\\ \frac{1-\beta }{1-\delta } & \frac{\beta -1}{1-\delta } \end{array}\right]$ is negative definite.

We assume $C_{1}=\left|\frac{\partial^{2}\Pi_{M}^{T\;}}{\partial p_{r}{}^{2}}\right|$, $C_{2}=\left|\begin{array}{cc} \frac{\partial^{2}\Pi_{M}^{T\;}}{\partial p_{r}{}^{2}} & \frac{\partial^{2}\Pi_{M}^{T}}{\partial p_{r}\partial \omega_{n}}\\ \frac{\partial^{2}\Pi_{M}^{T}}{\partial \omega_{n}\partial p_{r}} & \frac{\partial^{2}\Pi_{M}^{T\;}}{\partial \omega_{n}{}^{2}} \end{array}\right|$, where $C_{1}=\frac{\left(1-\beta \right)\left(\delta -2\right)}{\delta \left(1-\delta \right)}-2\beta <0$, $C_{2}=\frac{2\left(1-\beta \right)\left(1-\beta +\beta \delta \right)}{\delta \left(1-\delta \right)}>0$. In other words, the Hessian matrix is negative definite.

Therefore, $\Pi_{M}^{T}\left(p_{r},\omega_{n}\right)$ is a concave function of $p_{r}$ and $\omega_{n}$. Let $\partial \Pi_{M}^{T}/\partial p_{r}=0$ and $\partial \Pi_{M}^{T}/\partial \omega_{n}=0$, we can obtain the optimal decisions $\omega_{n}^{T}{}^{*}=\left(c+t+1\right)/2+Y$ and $p_{r}^{T}{}^{*}=\left(\delta +\alpha t\right)/2+Y$. Substituting the above values into the retailer’s best response, we get $p_{n}^{T}{}^{*}=\left[3-\delta +c+\left(1+\alpha \right)t\right]/4+Y$. From (9), we get:

$\begin{array}{c} \frac{\partial SW^{T}\left(t\right)}{\partial t}=\\ \frac{\left[\left[\left(4\delta^{2}-7\delta +4\right)\beta +3\delta -4\right]\alpha^{2}+\delta \left(\beta -1\right)\left(1-2\alpha \right)\right]t+\left[3\delta \left(\alpha -1\right)\left(\delta -1+c\right)-4v\left[\left(2\delta^{2}-3\delta +2\right)\alpha^{2}-2\alpha \delta +\delta \right]\right]\beta +\left[4v\left[\left(2-\delta \right)\alpha^{2}-2\alpha \delta +\delta \right]-3\delta \left(1-c-\delta +\alpha c\right)+\alpha \delta \left(\delta -1\right)\right]}{16\delta \left(1-\delta \right)} \end{array}$

$\frac{\partial^{2}SW^{T}\left(t\right)}{\partial t^{2}}=\frac{\left[\left(4\delta^{2}-7\delta +4\right)\beta +3\delta -4\right]\alpha^{2}+\delta \left(\beta -1\right)\left(1-2\alpha \right)}{16\delta \left(1-\delta \right)}$.

Since the second-order derivatives $\partial^{2}SW^{T}\left(t\right)/\partial t^{2}<0$, $SW^{T}\left(t\right)$ is a concave function of μ. Let $\frac{\partial SW^{T}\left(t\right)}{\partial t}=0$, and we can obtain the optimal unit of carbon tax:

$t^{*}=\frac{-\left[3\delta \left(\alpha -1\right)\left(\delta -1+c\right)-4v\left[\left(2\delta^{2}-3\delta +2\right)\alpha^{2}-2\alpha \delta +\delta \right]\right]\beta +\left[4v\left[\left(\delta -2\right)\alpha^{2}+2\alpha \delta -\delta \right]+3\delta \left(1-c-\delta +\alpha c\right)-\alpha \delta \left(\delta -1\right)\right]}{\left[\left(4\delta^{2}-7\delta +4\right)\beta +3\delta -4\right]\alpha^{2}+\delta \left(\beta -1\right)\left(1-2\alpha \right)}$.

To ensure a non-negative unit of carbon tax, we have:

When $0<v<v_{0}$, if $\beta >\beta_{v}$, then $t=t^{*}$, if $\beta <\beta_{v}$, then t=0;

when $v_{0}<v$, if $\beta >\beta_{v}$, then t=0, if $\beta <\beta_{v}$, then $t=t^{*}$.

where $v_{0}=\frac{3\delta \left(\alpha -1\right)\left(\delta +c-1\right)}{4\left[\left(2\delta^{2}-3\delta +2\right)\alpha^{2}-2\alpha \delta +\delta \right]}$, $\beta_{v}=\frac{-4v\left[\left(2-\delta \right)\alpha^{2}-2\alpha \delta +\delta \right]+3\delta \left(1-c-\delta +\alpha c\right)+\alpha \delta \left(\delta -1\right)}{3\delta \left(\alpha -1\right)\left(\delta -1+c\right)+4v\left[\left(2\delta^{2}-3\delta +2\right)\alpha^{2}-2\alpha \delta +\delta \right]}$

## Appendix C

**Proof of Proposition 3:**

$E^{S}{}^{*}-E^{Z}{}^{*}=\frac{\left(\beta -1\right)\left(\delta -2\alpha +\alpha \delta \right)\mu +2\alpha \beta \delta \left(1-\delta \right)\mu }{4\delta \left(1-\delta \right)}$.

If $\frac{\delta }{2-\delta }<\alpha <1$, we can obtain δ−2α+αδ<0; therefore, $E^{S}{}^{*}-E^{Z}{}^{*}>0$; if $0<\alpha <\frac{\delta }{2-\delta }$, to prove $E^{S}{}^{*}>E^{Z}{}^{*}$, we have to show that (β−1)(δ−2α+αδ)μ+2αδ(1−δ)μβ>0, namely, $\beta >\beta_{a}$, and conversely, if $E^{S}{}^{*}<E^{Z}{}^{*}$, then $\beta <\beta_{a}$.

$E^{Z}{}^{*}-E^{T}{}^{*}=\frac{\left(\beta -1\right)\left[\left(\delta -2\right)\alpha^{2}+2\alpha \delta -\delta \right]t+2\alpha^{2}\beta \delta t\left(1-\delta \right)}{4\delta \left(1-\delta \right)}$. We assume that $f\left(\alpha \right)=\left(\delta -2\right)\alpha^{2}+2\alpha \delta -\delta$, where $f\left(\frac{\delta }{2-\delta }\right)=\frac{2\delta \left(\delta -1\right)}{2-\delta }<0$. We can obtain f(α)<0. Therefore, $E^{Z}{}^{*}>E^{T}{}^{*}$.

$E^{S}{}^{*}-E^{T}{}^{*}=\frac{\left(\beta -1\right)\left[\left(\delta -2\alpha +\alpha \delta \right)\left(\mu +\alpha t\right)+\delta \left(\alpha -1\right)t\right]+2\alpha \delta \left(1-\delta \right)\left(\mu +\alpha t\right)\beta }{4\delta \left(1-\delta \right)}$.

If $\frac{\delta }{2-\delta }<\alpha <1$, we can obtain $E^{S}{}^{*}-E^{T}{}^{*}>0$; if $0<\alpha <\frac{\delta }{2-\delta }$, and (δ−2α+αδ)(μ+αt)+δ(α−1)t<0, namely, $\frac{t}{\mu }>\frac{\delta -\left(2-\delta \right)\alpha }{\left(2-\delta \right)\alpha^{2}-2\alpha \delta +\delta }$; then $E^{S}{}^{*}-E^{T}{}^{*}>0$; if $0<\alpha <\frac{\delta }{2-\delta }$, and $\frac{t}{\mu }\leq \frac{\delta -\left(2-\delta \right)\alpha }{\left(2-\delta \right)\alpha^{2}-2\alpha \delta +\delta }$, to prove $E^{S}{}^{*}>E^{T}{}^{*}$, we have to show that (β−1)[(δ−2α+αδ)(μ+αt)+δ(α−1)t]+2αδ(1−δ)(μ+αt)β>0, namely; $\beta >\beta_{0}$, conversely, if $E^{S}{}^{*}<E^{T}{}^{*}$,then $\beta <\beta_{0}$.

To sum up, we can obtain the above Proposition 3.

**Proof of Proposition 6:**

$SW^{T}\left(t^{*}\right)-SW^{T}\left(0\right)>0$, namely, $SW^{T}{}^{*}>SW^{Z}{}^{*}$.

$SW^{S}{}^{*}-SW^{Z}{}^{*}=\frac{{\left[-4\beta v\left(\delta -2\alpha +3\alpha \delta -2\alpha \delta^{2}\right)+3\delta \beta \left(1-\delta -c\right)+4\left(\delta -2\alpha +\alpha \delta \right)+\delta \left(1-\delta +3c\right)\right]}^{2}}{32\delta \left(\delta -1\right)\left[\left(\beta -1\right)\left(4-3\delta \right)+4\beta \delta \left(\delta -1\right)\right]}>0$, thus, $SW^{S}{}^{*}>SW^{Z}{}^{*}$.

$SW^{S}{}^{*}-SW^{T}{}^{*}=\frac{\left(\beta -1\right)\left[\left(1-\delta \right)F_{1}\beta^{2}+F_{2}\beta +F_{3}\right]}{8\left[\left(\beta -1\right)\left(4-3\delta \right)+4\beta \delta \left(\delta -1\right)\right]\left[\left(\beta -1\right)\left(\delta +4\alpha^{2}-2\alpha \delta -3\alpha^{2}\delta \right)+4\alpha^{2}\beta \delta \left(\delta -1\right)\right]}$.

Let $F_{1}\beta^{2}+F_{2}\beta +F_{3}={\left[\begin{array}{c} \beta^{2}\\ \beta \\ 1 \end{array}\right]}^{T}\left[\begin{array}{ccc} F_{11} & F_{12} & F_{13}\\ F_{21} & F_{22} & F_{23}\\ F_{31} & F_{32} & F_{33} \end{array}\right]\left[\begin{array}{c} v^{2}\\ v\\ 1 \end{array}\right]$, where

$F_{11}=16\left(\delta -1\right)\left[\left(1+\delta^{2}\right)\left(2\alpha -3\right)\alpha^{2}-\delta \left(3\alpha^{3}-3\alpha^{2}-3\alpha +1\right)\right]$,

$F_{12}=12\left(1-\delta \right)\left(c+\delta -1\right)\left(2\delta +4\alpha^{2}-5\alpha \delta -5\alpha^{2}\delta +4\alpha^{2}\delta^{2}\right)$,

$F_{13}=9\delta \left(\delta -1\right)\left(2\alpha -1\right){\left(c+\delta -1\right)}^{2}$,

$F_{21}=16\left[\delta \left(\delta -2\right)\left(3\alpha^{3}-3\alpha^{2}-3\alpha +1\right)\right]$,

$\begin{array}{cc} F_{22}= & -4[\left(15\alpha^{2}+11\alpha -6\right)\delta^{3}+\left(15\alpha c-6c-33\alpha +15\alpha^{2}c-47\alpha^{2}+16\right)\delta^{2}+2(13\alpha +\\ & 6c-15\alpha c-15\alpha^{2}c+27\alpha^{2}-6)\delta +24\alpha^{2}\left(c-1\right)], \end{array}$

$F_{23}=-3\delta \left(c+\delta -1\right)\left(4\alpha +6c+7\delta -12\alpha c-3\delta c-2\alpha \delta^{2}-3\delta^{2}+6\alpha \delta c-6\right)$,

$F_{31}=16\left[\left(\alpha^{3}-3\alpha +1\right)\delta -2\alpha^{3}+3\alpha^{2}\right]$,

$F_{32}=4\left[-\left(5\alpha^{2}+7\alpha -4\right)\delta^{2}+\left(-15\alpha c+6c+11\alpha -3\alpha^{2}c+15\alpha^{2}-6\right)\delta +12\alpha^{2}\left(c-1\right)\right]$,

$F_{33}=\left(4\alpha +7\right)\delta^{3}+2\left(6c-5\alpha -3\alpha c-8\right)\delta^{2}+3\left(2\alpha -6c+4\alpha c-6\alpha c^{2}+3c^{2}+3\right)\delta$.

$f_{1}\left(\delta \right)=\left(\beta -1\right)\left(\delta +4\alpha^{2}-2\alpha \delta -3\alpha^{2}\delta \right)+4\alpha^{2}\beta \delta \left(\delta -1\right)$ is a convex function of δ, $f_{1}\left(\delta \right)<0.$

$f_{2}\left(\delta \right)=\left(1+\delta^{2}\right)\left(2\alpha -3\right)\alpha^{2}-\delta \left(3\alpha^{3}-3\alpha^{2}-3\alpha +1\right)$ is a concave function of δ,$\;-1<f_{2}\left(\delta \right)<0$.

Thus, $F_{11}>0$. As a result,$\;F_{1}$ is a convex function of v. Let $F_{1}=0$, we can obtain

$v_{a}=\frac{-3\left(1-c-\delta \right)\left[2\delta +4\alpha^{2}-5\alpha \delta -5\alpha^{2}\delta +4\alpha^{2}\delta^{2}-\alpha \sqrt{\left(4\delta^{2}-7\delta +4\right)\left[\left(4\delta^{2}-7\delta +4\right)\alpha^{2}-2\alpha \delta +\delta \right]}\right]}{8\left[\left(1+\delta^{2}\right)\left(2\alpha -3\right)\alpha^{2}-\delta \left(3\alpha^{3}-3\alpha^{2}-3\alpha +1\right)\right]}$,

$v_{b}=\frac{-3\left(1-c-\delta \right)\left[2\delta +4\alpha^{2}-5\alpha \delta -5\alpha^{2}\delta +4\alpha^{2}\delta^{2}+\alpha \sqrt{\left(4\delta^{2}-7\delta +4\right)\left[\left(4\delta^{2}-7\delta +4\right)\alpha^{2}-2\alpha \delta +\delta \right]}\right]}{8\left[\left(1+\delta^{2}\right)\left(2\alpha -3\right)\alpha^{2}-\delta \left(3\alpha^{3}-3\alpha^{2}-3\alpha +1\right)\right]}$.

Let $v_{1}=\min\left\{v_{a},v_{b}\right\}$,$\;v_{2}=\max\left\{v_{a},v_{b}\right\}$. When $v_{1}<v<v_{2}$, $F_{1}<0$, $\left(1-\delta \right)F_{1}\beta^{2}+F_{2}\beta +F_{3}$ is a concave function of β. Making it equal to zero, we can obtain:

$\beta_{a}=\frac{-D_{2}-\delta \left|3c+4v+3\delta -4\alpha v-3\right|\times \sqrt{\left|G\right|}}{2\left(1-\delta \right)\left[\left(\left(4\delta^{2}-7\delta +4\right)\beta +3\delta -4\right)\alpha^{2}+\delta \left(\beta -1\right)\left(1-2\alpha \right)\right]}$,

$\beta_{b}=\frac{-D_{2}+\delta \left|3c+4v+3\delta -4\alpha v-3\right|\times \sqrt{\left|G\right|}}{2\left(1-\delta \right)\left[\left(\left(4\delta^{2}-7\delta +4\right)\beta +3\delta -4\right)\alpha^{2}+\delta \left(\beta -1\right)\left(1-2\alpha \right)\right]}$.

Let $\beta_{1}=\min\left\{\beta_{a},\beta_{b}\right\}$,$\;\beta_{2}=\max\left\{\beta_{a},\beta_{b}\right\}$. If $\beta_{1}<\beta <\beta_{2}$, $SW^{S}{}^{*}<SW^{T}{}^{*}$; if $\beta <\beta_{1}$, or $\beta <\beta_{2}$, then $SW^{S}{}^{*}>SW^{T}{}^{*}$.

When $v<v_{1}$, or $v>v_{2}$, $F_{1}>0$, $\left(1-\delta \right)F_{1}\beta^{2}+F_{2}\beta +F_{3}$ is a convex function of β. If $\beta_{1}<\beta <\beta_{2}$, $SW^{S}{}^{*}>SW^{T}{}^{*}$; if $\beta <\beta_{1}$, or $\beta <\beta_{2}$, then $SW^{S}{}^{*}<SW^{T}{}^{*}$.

Let $G={\left[\begin{array}{c} \delta^{4}\\ \delta^{3}\\ \delta^{2}\\ \delta \\ 1 \end{array}\right]}^{T}\left[\begin{array}{ccc} 0 & 0 & G_{13}\\ 0 & G_{21} & G_{23}\\ G_{31} & G_{32} & G_{33}\\ 0 & G_{42} & G_{43}\\ 0 & 0 & G_{53} \end{array}\right]\left[\begin{array}{c} v^{2}\\ v\\ 1 \end{array}\right]$, where:

$G_{13}={\left(2\alpha +3\right)}^{2}$,

$G_{21}=8\left(2\alpha^{3}-\alpha^{2}-8\alpha +3\right)$,

$G_{23}=-2\left(34\alpha -9c+12\alpha c+12\alpha^{2}c+16\alpha^{2}+7\right)$,

$G_{31}=16{\left(\alpha -1\right)}^{4}$,

$G_{32}=8\left(10\alpha +3c-12\alpha c+15\alpha^{2}c-6\alpha^{3}c+11\alpha^{2}-8\alpha^{3}-5\right)$,

$G_{33}=\left(36\alpha^{2}c^{2}+96\alpha^{2}c+96\alpha^{2}-36\alpha c^{2}+12\alpha c+128\alpha +9c^{2}-30c-7\right)$,

$G_{42}=32\alpha \left(2\alpha^{2}-4\alpha +1\right)$,

$G_{43}=-16\left(5\alpha -3\alpha c+6\alpha^{2}c+8\alpha^{2}-1\right)$,

$G_{53}=64\alpha^{2}$.
